# Cost-Effective Isolation of a Process Impurity of Pregabalin

**DOI:** 10.3797/scipharm.1501-16

**Published:** 2015-03-25

**Authors:** Lakkireddy Prakash, Malipeddi Himaja, Belly Ramakrishna Yadav, Arumalla Maheshwara Reddy

**Affiliations:** 1 Department of Analytical Research and Development, Dr. Reddy’s, Laboratories Ltd, IPDO, Hyderabad, 500072, Telangana, India; 2 Pharmaceutical Chemistry Division, School of Advanced Sciences, VIT University Vellore-632014, TN, India

**Keywords:** Pregabalin, Impurity, Isolation, Preparative HPLC, Flash chromatography, Simulating moving bed

## Abstract

Cost-effective isolation methods were developed on preparative HPLC, flash LC, and simulated moving bed (SMB) to prepare the process impurity, 3-(aminomethyl)-5-methylhex-4-enoic acid (4-ene impurity), of pregabalin. By a thorough experimental study on the different isolation techniques available, it was concluded that SMB was the most cost-effective. Hence, it was a continuous chromatography that utilized the advantage of SMB so that a high quantity of the impurity was generated in a short period of time. SMB was equipped with eight reversed-phased columns and was used to separate the process impurity of pregabalin. The effects of flow rate in zone 2 (Q2) and 3 (Q3), as well as switching time, on the operating performance parameters like purity, productivity, and desorbent consumption were studied. Operating conditions leading to more than 90% purity in the raffinate outlet stream were identified, together with those achieving optimal performance. All of these developed methods are novel, cost-effective, and can be applied to the isolation of other process- and stability-related impurities of pregabalin.

## Introduction

Pregabalin, (3*S*)-3-(aminomethyl)-5-methylhexanoic acid, is a novel and potent anticonvulsant agent for the treatment of epilepsy and pain [1]. It has also been found to be more active than gabapentin in preclinical models of epilepsy [2]. It has more potent and robust activity in various models of epilepsy, neuropathic pain, and anxiety [3]. It is also used with other medications to treat certain types of seizures [4]. Pregabalin may be used as a second-line medication in general anxiety disorder [5]. The presence of impurities in an active pharmaceutical ingredient (API) can have a significant impact on the quality and safety of the drug products. Therefore, it is necessary to study the impurity profile of the API to be used in the manufacturing of a drug product.

Impurity profiling is the combination of analytical activities with the aim of detecting, isolating, identifying, or elucidating the structure and quantitatively determining impurities in bulk drugs and pharmaceutical formulations. There is an ever-growing interest of scientific communities and industries in impurities present in APIs and in finished products. Different pharmacopoeias, such as the British Pharmacopoeia (BP), United States Pharmacopeia (USP), Indian Pharmacopoeia (IP), and various regulatory authorities like the USFDA and Canadian Drug and Health Agencies, are slowly incorporating limits to allowable levels of impurities present in the APIs or formulations. Also, the International Conference on Harmonization (ICH) has published certain guidelines on impurities in drug substances, products, and residual solvents [6–9].

Impurities could arise at any stage of synthetic steps, resulting in a process-related impurity. Impurities could also arise due to degradation of an API or drug product during storage or due to drug-excipient interactions during manufacturing, resulting in a degradation-related impurity. Analytical activities concerning impurities in drugs are among the most important issues in modern pharmaceutical analysis and the isolation activity is quite necessary and plays an important role in impurity profiling [10]. A list of methods that can be used for the isolation of impurities are solid-phase extraction methods, liquid-liquid extraction methods, accelerated solvent extraction methods, supercritical fluid extraction, column chromatography, flash chromatography, capillary electrophoresis (CE), gas chromatography (GC), thin-layer chromatography (TLC), high-performance thin-layer chromatography (HPTLC), preparative high-performance liquid chromatography (preparative HPLC), supercritical fluid chromatography (SFC), and simulated moving bed (SMB). A literature survey revealed that there was no complete synthetic method or isolation method available for the preparation of the 4-ene impurity of pregabalin. The present manuscript deals with the development of isolation methods for the 4-ene impurity of pregabalin by using different methods like preparative HPLC, flash chromatography, and SMB.

## Experimental

### Chemicals and Reagents

The pregabalin drug substance and its impurities were obtained from Dr. Reddy’s Laboratories Limited, Hyderabad, India. The HPLC grade acetonitrile, analytical grade NaH_2_PO_4_, and sodium hydroxide pellets were purchased from Merck, Mumbai, India. High-purity water was collected from a Millipore Milli-Q Plus water purification system (Millipore, Milford, MA, USA).

### Equipment

An Agilent 1200 series with a high-pressure liquid chromatographic instrument provided with a sample manager, photodiode array (PDA) detector, and a thermostatted column compartment were used. The output signals were monitored and processed using Empower 2 software. The Agilent 1200 series preparative HPLC system was equipped with an automated fraction collector and photodiode array detector. The data were collected and processed using Chemstation software. The output signals were monitored and processed also using Chemstation software. Flash chromatography experiments were performed on the Reveleris system (Grace, USA) and SMB trails were taken on the Octave Chromatography system (Semba Biosciences, Inc. Madison, USA). The pH of the solutions was measured by a pH meter (Mettler-Toledo, Switzerland).

### Chromatographic Conditions

#### Analytical HPLC

The analytical method was developed using an XBridge C_8_ (150 x 4.6 mm, 3.5 μm) column with a mobile phase containing a mixture of solvent A (0.01 M sodium dihydrogen phosphate monohydrate, pH-adjusted to 6.3 with diluted sodium hydroxide solution) and solvent B (mixture of acetonitrile and water 75:25 v/v) mixed in the ratio of 97:3 v/v. The mobile phase was filtered through nylon 0.45 mm membrane filters and degassed. The flow rate of the mobile phase was 0.8 mL/min. The column temperature was maintained at 25°C and the eluted compounds were monitored at the wavelength of 210 nm. The injection volume was 20.0 μL.

#### Preparative HPLC

The preparative isolation study was performed on an Agilent 1100 series preparative HPLC system which was equipped with an automated fraction collector and variable wavelength detector. The data were collected and processed using Chemstation software. Approximately 33 mg/mL of the sample was prepared to load onto the column. An XBridge™ Prep Shield RP18 (19 x 250 mm, 5.0 μm) column was employed for the isolation of the impurity. Mobile phase A consisted of Milli-Q water and mobile phase B contained acetonitrile. The gradient program (time (min) / %B) was set as 0.01/2, 2/2, 12/30, 13/2, and 15/2 with a flow rate of 10.0 mL/min and injection volume of 1500 µL. The column temperature was maintained at 25°C and the peaks were monitored at 210 nm. Milli-Q water was used as diluent for the sample preparation. The impurity fractions were collected separately from several injections and pooled separately. The pooled fraction was concentrated by using a Rotavapour (model: Heidolph Laboratory 4002 control) under high vacuum. The aqueous solutions were lyophilized (model: Virtis Advantage Plus) to solidify the impurities.

#### Flash Chromatography System

Isolation work was performed on the Reveleris flash chromatography system which was equipped with a fraction collector, UV, and ELSD detectors. Approximately 80 mg/mL of the sample was prepared to load onto the column. The Reveleris RP C_18_ (12 g, 40 μm) cartridge was employed for the isolation of the impurity. Mobile phase A contained Milli-Q water and mobile phase B contained acetonitrile and Milli-Q water in the ratio of 75:25 v/v. The gradient program (time (min) / %B) was set as 0.01/2, 8/4, 8.5/60, 11.5/60, 12.5/0, and 14/0 with a flow rate of 20.0 mL/min and injection volume of 5 mL. The peaks were monitored at 210 nm. Milli-Q water was used as diluent for sample preparation. The impurity fractions were collected separately from several injections and pooled separately. The pooled fraction was concentrated by using a Rotavapour (model: Heidolph Laboratory 4002 control) under high vacuum. The aqueous solutions were lyophilized (model: Virtis Advantage Plus) to solidify the impurities.

#### Simulated Moving Bed Chromatography

Isolation was performed by using the simulated moving bed chromatography technique on the Octave chromatography system (Semba Biosciences, Inc.) which was equipped with four binary pumps having a capacity of 1 mL to 100 mL. Approximately 15 mg/mL of the sample was prepared to load with a feed rate 1.5 mL/min onto the column. An Orpheus RP (300 mm x 22 mm i.d., 300.0 μm) column was employed for the isolation of the impurity. It consisted of a carousel of eight stainless steel columns connected to a single multi-function valve. The desorbent contained Milli-Q water and acetonitrile in the ratio of 97:3 v/v with a flow rate of 15.0 mL/min, the extract flow rate was 5 mL/min, and the raffinate flow rate was 11.5 mL/min. The column temperature was maintained at 25°C and the switching time was 1650 sec. Milli-Q water was used as diluent for the sample preparation. The separation activity was carried out by using an isocratic 3.2.3 mode. The raffinate fractions were collected separately from several cycles and pooled separately. The pooled fraction was concentrated by using a Rotavapour (model: Heidolph Laboratory 4002 control) under high vacuum. The aqueous solutions were lyophilized (model: Virtis Advantage Plus) to solidify the impurities.

### Sample Preparation

#### Analytical HPLC

A stock solution was prepared by dissolving 100 mg of crude pregabalin in 100 mL of Milli-Q water. Five mL of the above stock solution was diluted to 100 mL with Milli-Q water.

#### Preparative HPLC

Two g of crude pregabalin was dissolved in 60 mL of Milli-Q water (33 mg/mL).

#### Flash Chromatography System

An amount of 800 mg of crude pregabalin was dissolved in 60 mL of Milli-Q water (80 mg/mL).

#### Simulated Moving Bed Chromatography

An amount of 1500 mg of crude pregabalin was dissolved in 100 mL of Milli-Q water (15 mg/mL).

### Synthetic Scheme for the Preparation of the Impurity

**Fig. 1:**
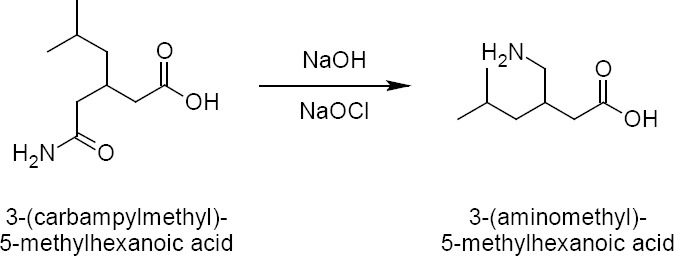
Synthesis of pregabalin

During the process development for the synthesis of pregabalin (Figure 1), HPLC analysis of crude pregabalin revealed one process impurity: the 4-ene impurity ranging from 0.1–2.0% in different batches. The purification step of the synthesis was carefully optimized to lower the impurities to <0.15%. According to ICH (International Conference on Harmonization) guidelines, the amount of an acceptable level for known impurities in a final drug candidate must be less than 0.15%. In order to meet the stringent regulatory requirements, the impurity needs to be identified and characterized. Hence, different batches of pregabalin were initially analysed by LCMS to provide parent ions at m/z 158 for the impurity of pregabalin and thus provide a basis for initial identification. Then we started working on the synthesis, followed by the isolation of the 4-ene impurity.

Two different processes were followed to prepare the impurity standard.

1. Linear synthetic approach
2. Synthetic enhancement followed by the isolation of the impurity

**Fig. 2:**
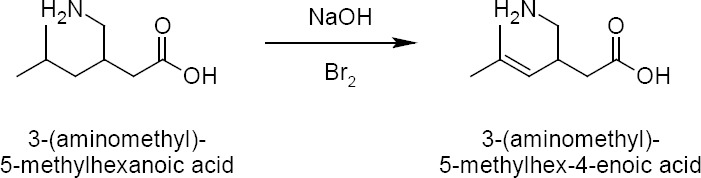
Synthetic enhancement of the impurity

**Fig. 3:**
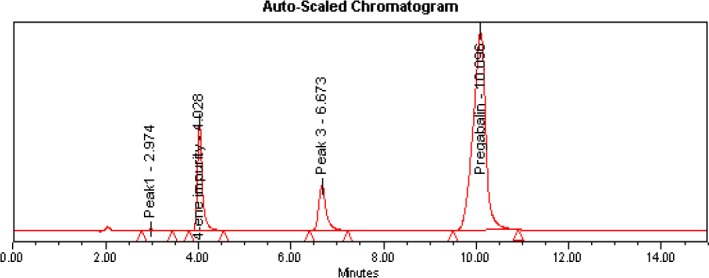
HPLC chromatogram of crude pregabalin

The linear approach was not successful in any synthetic experiment. So, the enhancement of the impurity was adopted by using highly basic conditions by taking pregabalin as the starting material (Figure 2). In this route, the impurity was enhanced up to 15.67% (Figure 3). Using regular chromatographic methods for the isolation of the impurity, we ended up with unsatisfactory results due to the absence of chromophoric groups present in the compound. Hence, isolation activity was taken up to get the pure impurity for characterisation followed by a validation study. Different techniques like preparative LC, flash chromatography, and simulated moving bed chromatography were used to carry out the isolation activity.

## Results and Discussion

### Isolation of the Impurity by Preparative HPLC

**Fig. 4:**
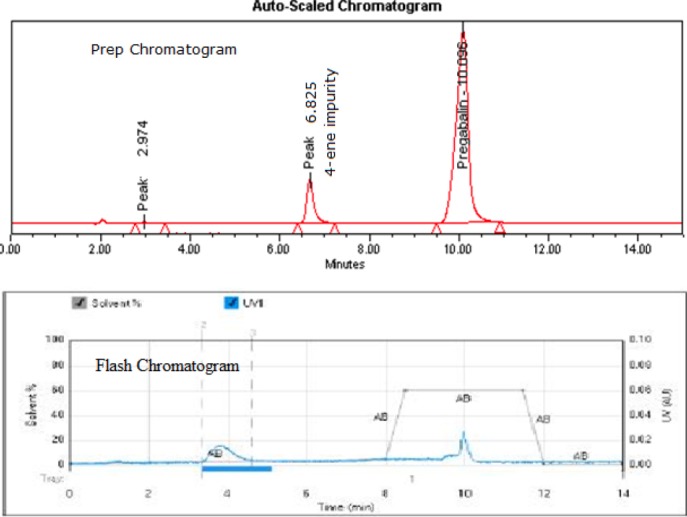
Prep HPLC and flash LC impurity isolation chromatograms

**Fig. 5:**
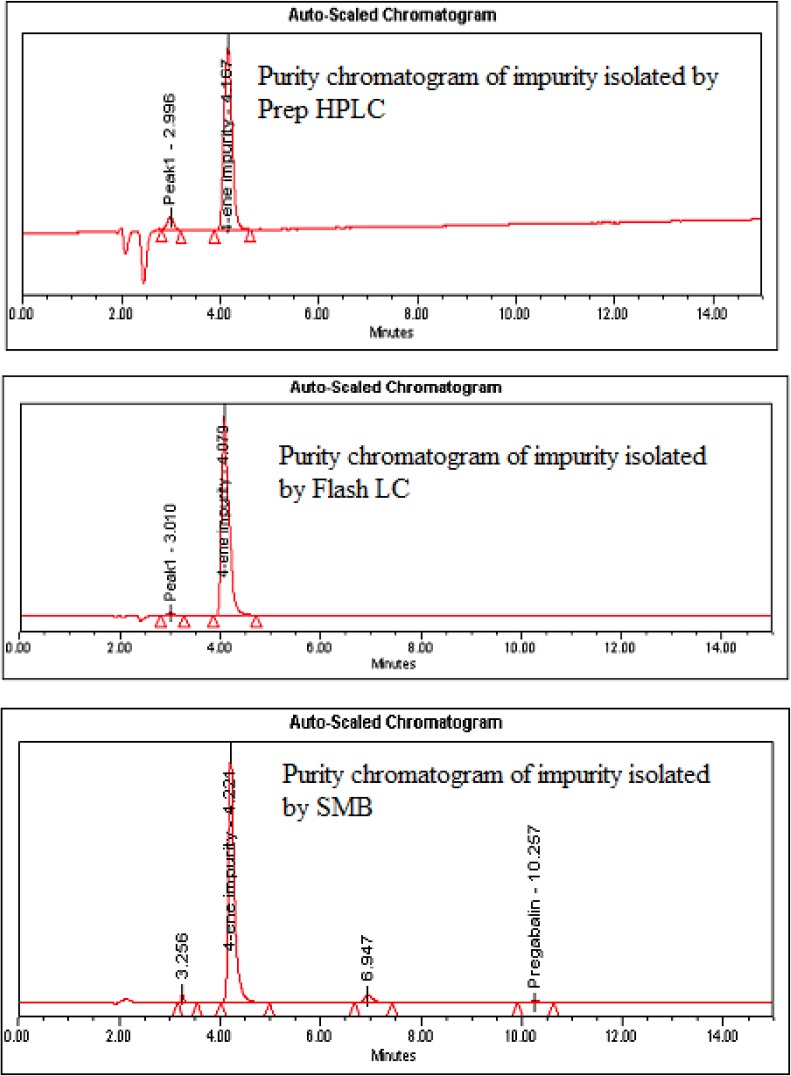
Purity chromatograms of the 4-ene impurity isolated by prep HPLC, flash LC, and SMB

A simple reversed-phase solvent system discussed in the “Experimental” section was used for isolating the impurity. Good resolution was achieved between pregabalin and the impurity (Figure 4). A sample loading study was carried out to inject the sample to its maximum extent, so 50 mg of crude pregabalin was injected per each injection and the impurity was collected on a threshold basis. The acetonitrile portion was evaporated at room temperature under high vacuum on a Heidolph Rotavapour model Laborota 4002. The aqueous layer was lyophilized to get the impurity in solid form. The purity of this impurity was found to be 98.86% (Figure 5) in the previously discussed analytical HPLC method. The yield of the impurity per each injection was calculated and found to be 65% (Table 1).

**Tab. 1:**
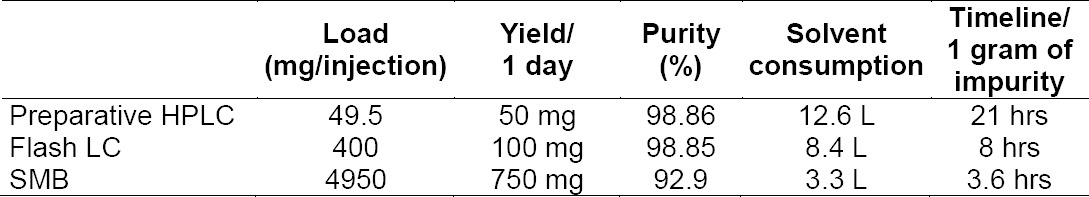
Summary of preparative HPLC, flash LC, and SMB isolation parameters

### Isolation of the Impurity by Flash Chromatography

About 80 mg of the crude pregabalin sample was loaded onto the RP C_18_ (12 g, 40 μm) cartridge and the peaks were eluted by using a simple reversed-phase solvent system discussed in the “Experimental” section. Under the discussed chromatographic conditions, pregabalin and the impurity were well-separated and enabled us to increase the load up to 400 mg (Figure 4) per each injection. Run time was also decreased compared to preparative HPLC. The organic portion was evaporated from the impurity fractions and the aqueous portion was kept for lyophilization to get the impurity in solid form. Purity and yield of the impurity were found to be 98.85% (Figure 5) and 75%, respectively, in flash chromatography (Table 1).

### Isolation of the Impurity by Simulated Moving Bed Chromatography

Simulated moving bed chromatography (SMBC) is a powerful approach to chromategraphic fractionation. SMBC emulates counter-current separation where the mobile phase flows in the opposite direction of the stationary phase. The stationary phase is represented by individual columns connected in a series, whereas the mobile phase is represented by inlet streams i.e. feed and desorbent and outlet streams, i.e. raffinate and extract, respectively. Valves between the columns are systematically switched open or closed at timed intervals (switch time) to introduce the inlet streams and withdraw the outlet streams between the separation zones, simulating counter-current movement of the columns. Separation occurs due to the differential migration of the feed mixture components through the column material. Components that interact more strongly with the column material are carried into the extract, whereas weaker-interacting components move into the raffinate. By adjusting the stream flow rates, the switch time, and the desorbent composition, a cycle is established in which feed and desorbent are continuously added and highly purified products are continuously recovered either in raffinate or in the extract [11–13].

The 3-2-3 designation refers to the number of columns in each SMBC zone. The zones are defined as follows:

- Zone 1: Between the desorbent inlet and extract outlet; where the more retained component is desorbed.
- Zone 2: Between the extract outlet and feed inlet; where the less retained component is desorbed and the more retained component is enriched.
- Zone 3: Between the feed inlet and raffinate outlet; where the more retained component is adsorbed and the less retained component is enriched and desorbed.

The aim of this work was to develop a novel isolation solution by using SMB and to investigate some important issues, including the influence of the switching time and the flow rates in Zones 2 and 3 on the performance of separation.

As discussed in the “Experimental” section, the column profile around the SMB was determined through the collection of samples from each position in the SMB. In a given switching time, the extract/raffinate solutions were collected. The sample was an average of the effluent from each column location, collected over the course of one carousel rotation. After the cyclic steady state was reached, the samples from the nodes of the columns were analyzed to determine the column profile.

All experiments were run at 30°C. The feed concentration was 15 mg/mL. The products of each run were collected during a few cycles of operation at steady-state conditions, which were reached after a few cycles of operation, where one cycle consisted of eight switches, eight being the number of columns. Five series of experimental runs have been performed with operating conditions in terms of flow rates (zone 1(Q1), zone 2(Q2), zone 3(Q3)), switch time *T*s (Table 2), together with the measured separation performance in terms of product purity, desorbent consumption, and productivity.

**Tab. 2:**
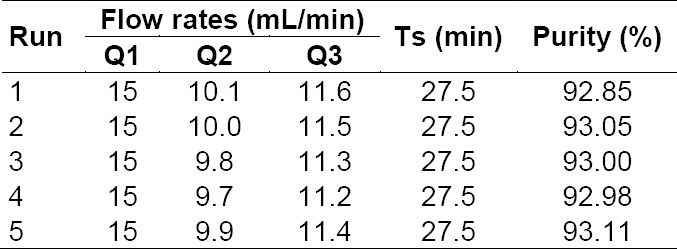
Operation conditions and separation performance of the runs

### Effect of Zone 2 and Zone 3 Flow Rates on Purity

**Fig. 6:**
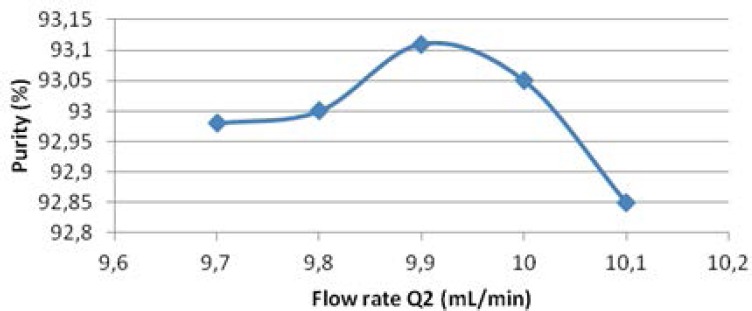
Effect of zone 2 flow rates on purity

**Fig. 7:**
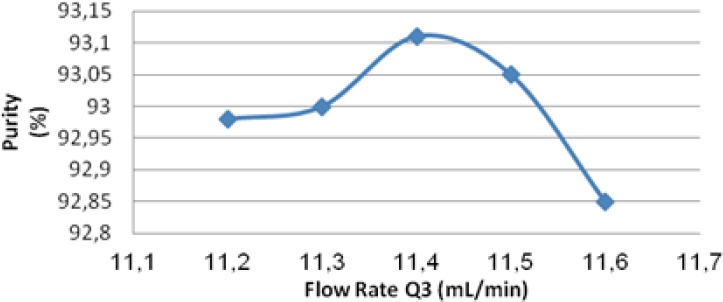
Effect of zone 3 flow rates on purity

The effect of flow rate of zone 2 (*Q*2) and 3 (*Q*3) on purity was listed in Table 2 and shown in Figure 6. With other flow rates constant, *Q*2 and *Q*3 related to each other. In other words, only one of these two flow rates could change independently. As shown in Table 2 and Fugure 7, the flow rate in zone 2 increased from 9.7 mL/min to 9.9 mL/min and the purity increased from 92.98% to 93.11%. As shown in Table 2 and Figure 7, the flow rate in zone 3 increased from 11.2 mL/min to 11.4 mL/min, while the purity increased from 92.9% to 93.1% (Figure 5).

### Effect of Switching Time on Purity

**Fig. 8:**
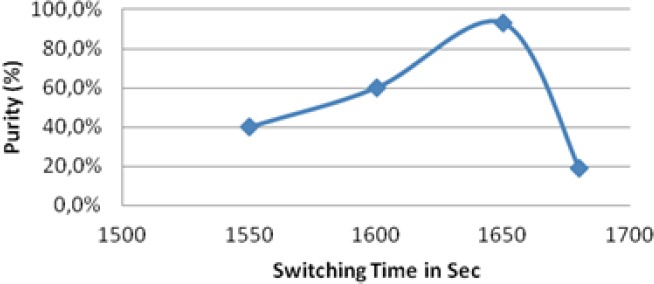
Effect of switching time on purity

The effect of switching time displayed in Figure 8 shows an increase in purity with increased switching time from 1550 sec to 1650 sec and a decrease in purity upon increasing the switching time more than 1650 sec.

## Conclusion

Cost-effective isolation methods were developed for the preparation of the process impurity of pregabalin on preparative HPLC, flash LC, and SMB, which was not possible to prepare by a synthetic route. The isolation parameters of SMB were optimized and the isolation conditions of different techniques were compared. The developed isolation methods can be tuned for the isolation of any other stability degradants.
